# Detection of p53 mutation and serum monitoring alert caused by Marek’s disease virus in poultry

**DOI:** 10.1186/s12917-020-02520-2

**Published:** 2020-08-24

**Authors:** Huixia Zhang, Mengda Liu, Hui Zhang, Shengliang Cao, Yue Li, Shengnan Jiang, Yinuo Song, Sidang Liu

**Affiliations:** 1 College of Animal Science and Veterinary Medicine, Shandong Agricultural University, 61 Daizong Street, 271018 Taian, Shandong China; 2 China Animal Health and Epidemiology Center, 369 Nanjing Road, 266032 Qingdao, Shandong China

**Keywords:** Marek’s disease, p53, P53 antibody

## Abstract

**Background:**

Marek’s disease (MD) is a chicken neoplastic disease, which brings huge economic losses to the global poultry industry. The wild type p53, a tumor suppressor gene, plays a key role in blocking cell cycle, promoting apoptosis, and maintaining the stability of the genome. However, the mutant p53 losses its tumor inhibitory role and become an oncogene when a mutation has happened.

**Results:**

The mutation rate of p53 was 60% in the experimentally and naturally infected chickens. The mutations included point-mutations and deletions, and mostly located in the DNA-binding domain. The mutated p53 was expressed in various tumor tissues in an infected chicken. The mutant P53 proteins were notably accumulated in the cytoplasm due to the loss in the function of nuclear localization. Unlike the study on human cancer, the concentrations of P53 in the serums of MD infected chicken were significantly lower than the control group.

**Conclusions:**

The p53 mutations were apparent in the development of MD. P53 and P53 antibody level in serum could be a useful marker in the diagnosis and surveillance of MD.

## Background

Marek’s disease (MD) is a lymphoproliferative neoplastic disease caused by the chicken Marek’s disease virus (MDV or named Gallid alphaherpesvirus 2). The infection caused by this virus may lead to lymphocyte proliferation, tumor formation, immunosuppression, paralysis, and mononuclear cell infiltration in peripheral nerves, gonads, and immune organs [1, 2]. As one of the most highly contagious tumor diseases in chickens, the data of OIE [3] reported by about half of the world has shown that this disease accounts for the loss of up to 1–2 billion US dollars annually in the global poultry industry [4]. Described often as the “guardian of the genome” [5] and the “cellular gatekeeper” [6], p53 is the most relevant and important tumor suppressor gene with the highest mutation frequency in human and animal tumor diseases [5]. The chicken p53 gene has a full-length open reading frame, 5’ and 3’ untranslated regions, and a polyadenylation signal, which encodes 367 amino acids. These amino acids share a 47% homology to the amino acids of human P53 [7].

p53 is divided into the wild type and the mutant type. The wild type is a normal tumor suppressor gene, while the mutant p53 is an oncogene transformed from a tumor suppressor gene due to spatial conformational change. As a result, the mutant P53 protein losses its ability in regulating cell growth, apoptosis, and DNA repair [8] which often a prerequisite for tumorigenesis and disease progression [9]. The proportion of p53 mutations in tumor tissue varies between 10–100% [9].

The wild type P53 has a very short half-life while the mutant P53 protein is prolonged. This abnormality of P53 can lead to the accumulation of P53 antibody in serum as well as the P53 protein in tumor tissue [10]. Several studies have demonstrated that the P53 antibody plays a predictive role in tumorigenesis and its manifestation in serum is an early event in the development of malignant tumors in humans [11–13]. The level of P53 antibody in serum correlates significantly with common clinical neoplastic diseases, but it has been barely detectable in the serum of healthy subjects [14]. This correlation may also exist in chicken and currently, there is a knowledge gap concerning the role of mutant p53 in Marek’s disease in chicken. Therefore, this study aimed to investigate the role of p53 as a tumor marker in assisting the clinical diagnosis and prognosis of MD.

## Results

### Histopathological and immunohistochemical analysis of p53 expression

**Fig. 1:**
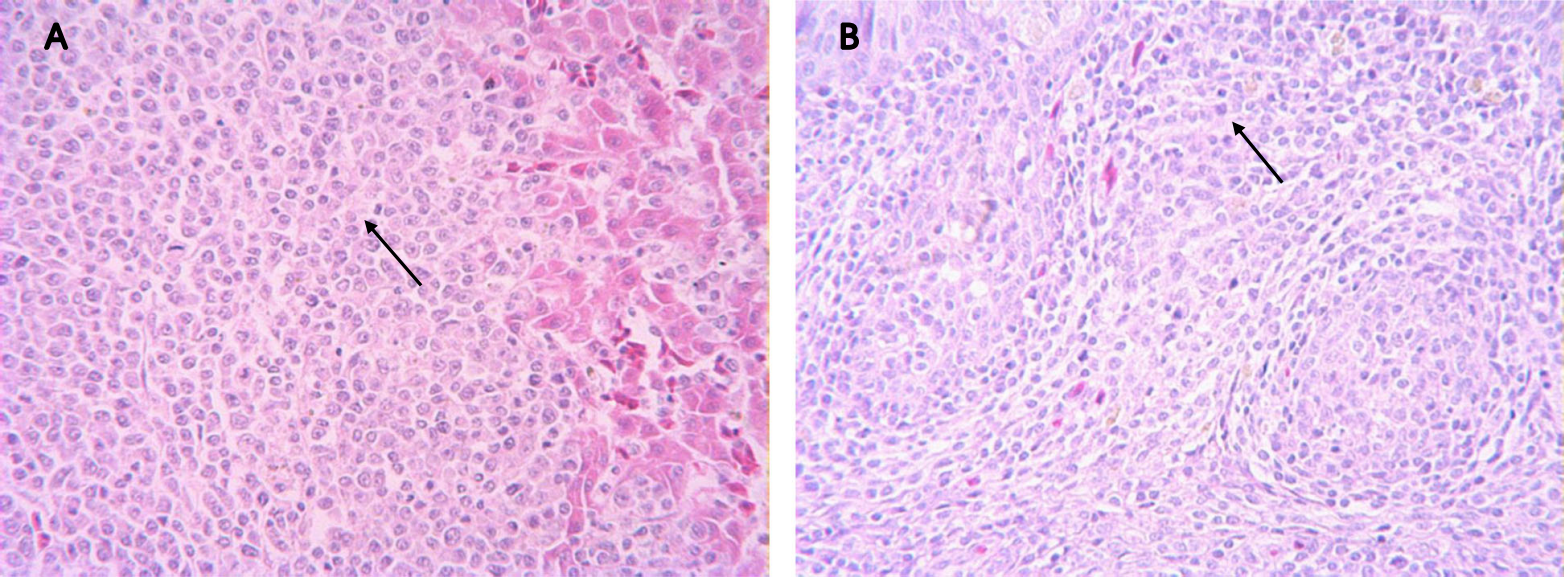
Histopathological observation of diseased chickens infected with MDV. **a** Different sizes and shapes of focal infiltration and hyperplasia of lymphoid tumor cells were observed in the liver tissues (HE, 400×). **b** Diffuse infiltration and proliferation of lymphoid tumor cells between the follicles were observed in the bursa of Fabricius (HE, 400×)

**Fig. 2:**
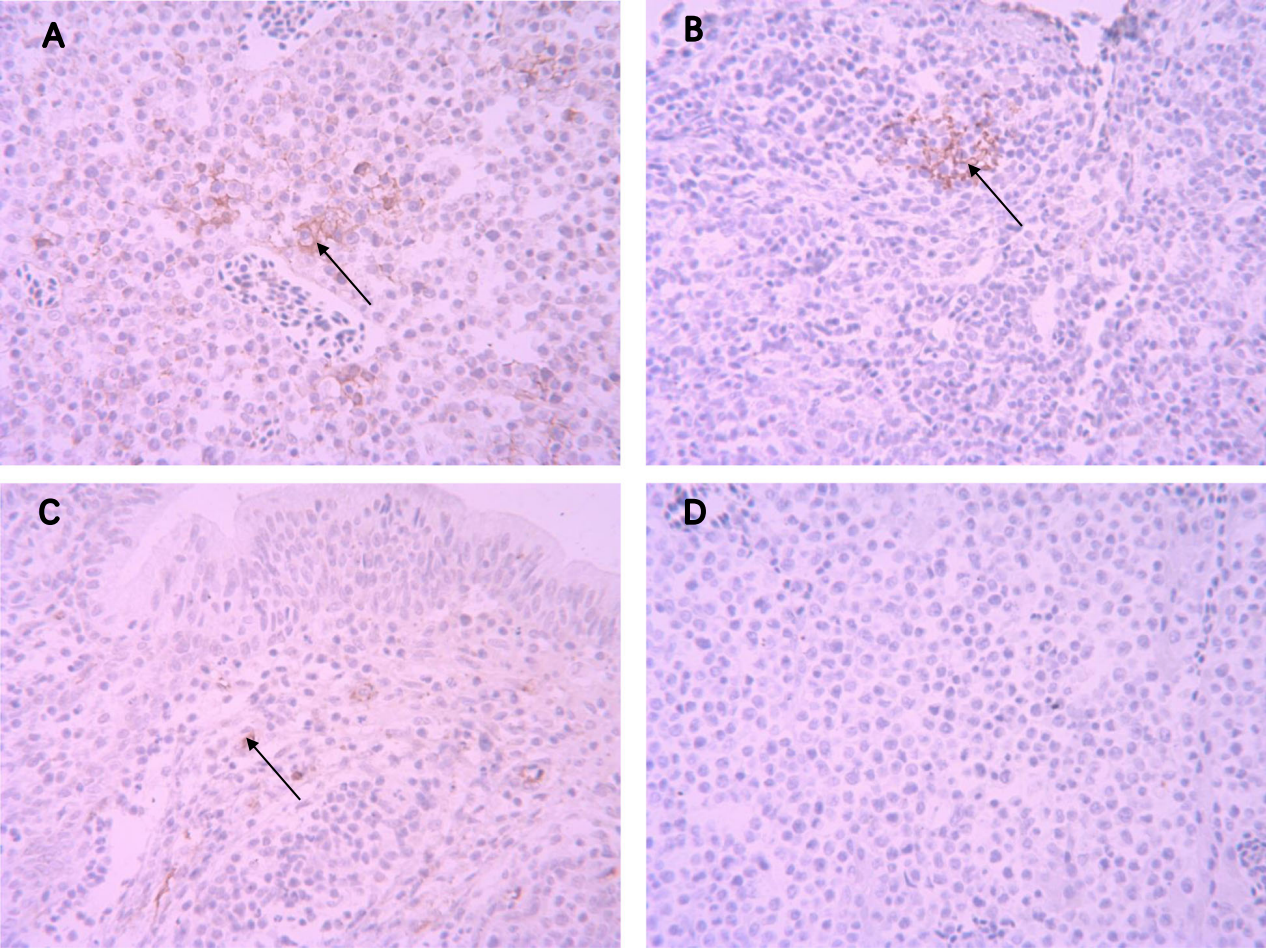
Immunohistochemical staining of p53 in infected chickens. **a** Liver - lymphoid tumor cells with cytoplasm expression (HE, 400×). **b** Spleen - lymphoid tumor cells with cytoplasm expression (HE, 400×). **c** Bursa of Fabricius - lymphoid tumor cells with cytoplasm expression (HE, 400×). **d** Liver - Negative controls were incubated with PBS (HE, 400×)

Histopathological examination revealed the evidence of liver cells undergoing necrosis in the infected SPF chicken. Such liver tissues demonstrated focal infiltration and hyperplasia of lymphoid tumor cells (Fig. 1a). In addition, the lymphoid follicle in the bursa of Fabricius was atrophied; diffuse infiltration and proliferation of lymphoid tumor cells between the follicles were observed (Fig. 1b). In the livers of the clinically infected chickens, the cytoplasm of the lymphoid tumor cells underwent multifocal proliferation, which was stained brown by the immunohistochemical (IHC) (Fig. 2a). Positive staining was also detected in the tumor cells in the spleen and the bursa of Fabricius tissues (Fig. 2b and c). In contrast, no positive stained cells were observed in the control group (Fig. 2d).

### Mutations in the p53

The mutation rate of p53 was 60% in the infected poultry. There were two types of p53 mutations (deletions and point mutations) detected, which was consistent with the results reported in a previous study [15]. Among the 12 mutated p53 genes, the base sites with high mutation frequency were 651, 786, 828, 864, and 879. There was no mutation found in the control group. Most of the mutations were located in the core domain and the C-terminal domain. The mutation analyses were shown in Table 1.

**Table 1:** Primers used toamplify chicken p53 cDNA

Primer	Nucleotide sequence	Annealing temperature (℃)	Size of fragments amplified(location)
p53-1	5’-GTGGCCGTCTATAAGAAATCAGA-3’	56	687 bp (496-1182)
p53-2	5’-AAAAAGGGGGCGTGGTCAGT-3’		

### P53 antigen and P53 antibody levels for MD

**Fig. 3:**
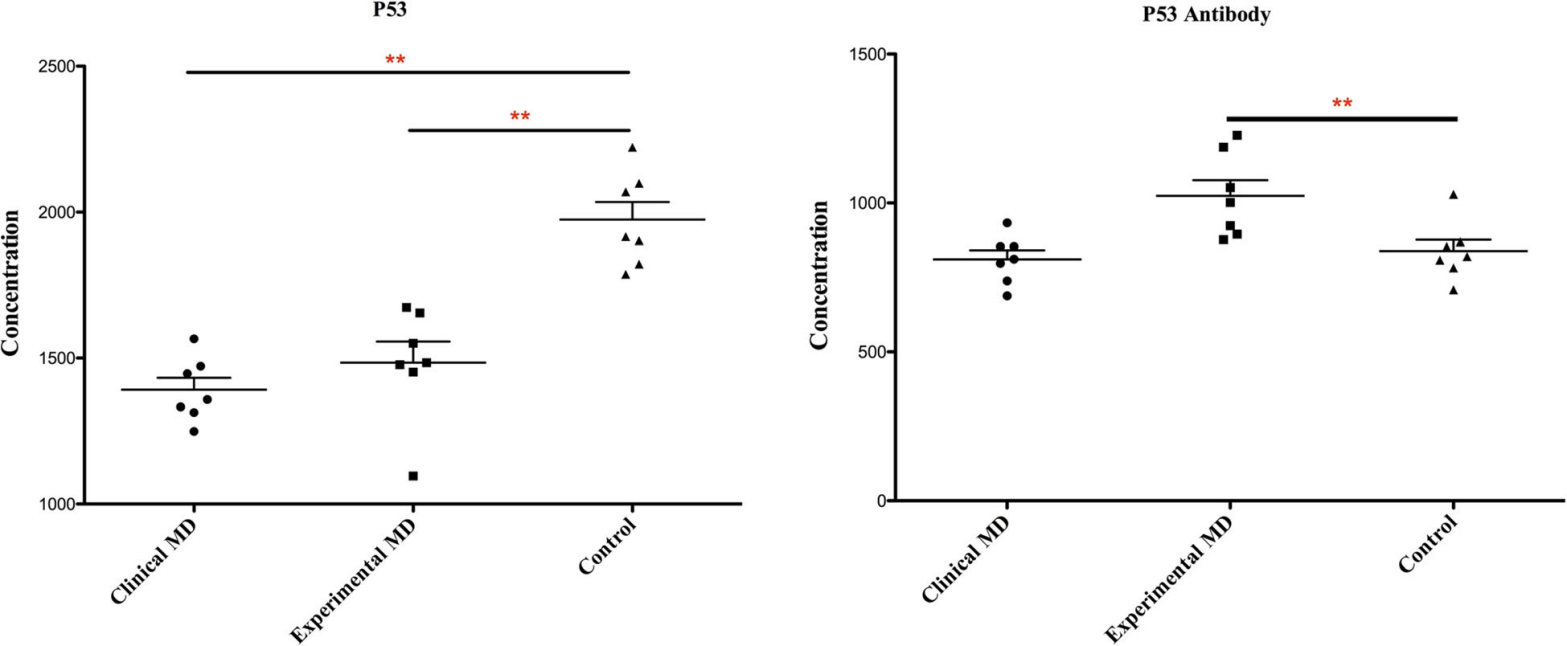
Levels of p53 antigen and antibody in the serum of study groups. The Y-axis represented the concentrations of antigen or antibody and the X-axis represented the different groups of samples. Each group consisted of seven samples. Statistical significance was designated as *p < 0.05 or ** *p* < 0.01

The concentrations of the P53 antigen of the clinical and experimental MDV-infected group were significantly lower than the control group. However, the P53 antibody levels in the experimental infection group were significantly higher than the control group. These analyses were shown in Fig. 3.

## Discussion

The human P53 is widely acknowledged as an intra-nuclear phosphorylated protein. The wide type P53 normally exists in the nucleus for an extremely short period [16]. On the other hand, the mutated P53 has a prolonged half-life to 20–40 h as it is not digested quickly and therefore accumulates inside tumor cells [17]. This allows the detection of mutant P53 protein via IHC, for which IHC has now become an important modality for the detection of various tumor biomarkers. P53 protein represents an effective substitute marker for TP53 mutation status [10] but at present, P53 IHC is mainly applied for tumor diseases in humans. P53 IHC allows the assessment of the stage and grade of cancers, with only a few studies reported in poultry tumor diseases.

In this study, the immunohistochemical analysis revealed that P53 was present in the liver, spleen, and the bursa of Fabricius of infected chicken with MDV. Interestingly, P53 was notably expressed in the cytoplasm. The potential explanation of this phenomenon could be that the mutated P53 protein lost its function in nuclear localization and eventually accumulated in the cytoplasm. Furthermore, p53 has been regarded as the “hotspots”, given that p53 mutations have been frequently detected in a variety of tumors [18]. In this study, several types of p53 mutations were demonstrated in natural and experimental infections of MDV. The five most frequent mutation sites were 651, 786, 828, 864, and 879. The altered codons of mutant p53 compared with the wild-type were 217 (ACG-ACA), 262 (GCA-GCC), 276 (CGC-CGG), 288 (GCC-GCA), and 293 (ACC-ACA), but these were all synonymous mutations.

In our study, a short form of p53 transcript was detected in a clinical case. The deleted sequence was located at the 631–677 bp in the open reading frame of the reference sequence, resulting in missense mutations from the position 210 to the termination. The short form of p53 transcript was also found in the cases infected by avian leukosis virus [15]. A series of missense mutations from the position 163 to the termination due to a base deletion was found in an experimentally infected chicken. In addition, this study found that the p53 was mutated in 60% of poultry oncology, with the mutation region concentrated mainly in the DBD. The DBD allows the specific recognition of target sequences [19]. Once the p53 is deleted or point mutations in this region occurs, it may affect the formation of tetramers, which in turn leads to the conversion of wild type P53 into a mutant type, resulting in the loss of normal function.

The conformation of mutant P53 extends its half-life to several hours in humans. The accumulated mutant P53 then acts as a target antigen which elicits an autoimmune response [20]. However, in our study, the levels of serum P53 antigen in clinical and experimental MDV-infected groups were significantly lower than the control group, contrary to the findings in human cancer research. Only the serum P53 antibody concentrations of the experimental infected group were significantly higher than the control group. This might be that P53, as a tumor suppressor, was largely consumed in response to the occurrence of MD. Moreover, tumorigenesis promoted mutation of p53 and further reduced P53 concentration.

## Conclusions

Our study revealed p53 was mutated and expressed in MDV infection, which suggested that these mutations were playing an important role in the development of MD. The mutant p53 was expressed in the tumor cells of various tissues of the infected chicken. Mutant P53 protein lost its nuclear localization function and transferred from the nucleus to the cytoplasm. Unlike studies in human cancer, the concentration of P53 was significantly lower in the natural and experimental MDV infection group. Our results may provide novel innovations for the diagnosis and monitoring of MD in poultry.

## Methods

### Natural infection of MDV in chickens

Seven 160-day-old egg-laying hens were obtained from a local flock. These hens were confirmed to have acquired MDV infection naturally with the absence of other common viral diseases by PCR detection. Their serums were collected for the detection of the P53 antigen and antibody. Their tissue samples, including liver, spleen, pancreas, and bursa of Fabricius, were collected and fixed with 4% formaldehyde for 48 h before histopathological and immunohistochemical studies.

### Experimental infection of chickens with MDV

The number of experimental animal was referred to the number of clinical samples. Twenty 1-day-old SPF chickens were obtained from the Shandong Academy of Agricultural Sciences Poultry Institute SPF Chicken Research Center (Jinan, China). They were randomly divided into two equal groups as an infection group and a control group. The two groups of chickens were raised separately in isolators with filtered air under positive pressure. On the first day of the experiment, each of the chicken in the infection group was inoculated intra-abdominally (i.a.) with 2,000 plaque-forming units (PFU) of vvMDV (GX0101), which normally cause tumors at the tenth week. The chickens in the control group were inoculated with PBS. In the tenth week, serums were collected from seven chickens in each group. The experiment ended after ten weeks and all chickens were put into a euthanasia box. Thirty percent of carbon dioxide (CO_2_) by volume was infused into the euthanasia box per minute until all chickens lost their consciousness. Then, the CO_2_ flow rate was increased to 100% for one minute and the euthanasia box was kept airtight for ten minutes. When all chickens were confirmed dead, they were dissected and their livers and spleens were collected for histopathology and immunohistochemistry study.

### Histopathology and Immunohistochemistry

The fixed liver, spleen, pancreas, and bursa of Fabricius were dehydrated, waxed, and cut into 3 µm slices, followed by Haematoxylin and eosin (H-E) staining for a histopathology examination. In IHC processing, tissues were cut into sections of 3 µm thickness and mounted on microslides treated with 0.1% poly-L-lysine. The sections were deparaffinized in xylene and rehydrated in a graded series of ethanol solutions into PBS, then were pre-treated in citrate buffer (0.01 mol/L; pH 6.0; 100 °C) for antigen retrieval by microwaving and cooled at room temperature for 20 min [21]. Endogenous alkaline peroxidase was quenched with 3% hydrogen peroxide solution in methanol for 30 min [22]. Nonspecific antibody binding sites were obliterated by incubating the sections with 5% fetal bovine serum for 60 min at room temperature. Following this, the anti-P53 polyclonal antibody (BOSTER, China) as a primary antibody was diluted into 1:200 and immersed overnight at 4 °C in a black humid chamber. The secondary antibodies were HRP (horseradish peroxidase)-conjugated goat anti-mouse IgG (CWBIO, China). Immunoreactivity was then visualized with diaminobenzidine (DAB) staining. The sections were counterstained with hematoxylin and mounted. Negative controls were incubated with PBS instead of the primary antibody in the immunohistochemical analysis.

### Total RNA isolation and reverse transcription

The total RNA of liver and spleen tissue samples (50 mg/tissue) were isolated by using TRIzol reagent (Takara, Japan) according to the manufacturer’s instructions. Extracted total RNA (1ug) was reverse transcribed to cDNA with PrimeScript™ RT Reagent Kit (Roche, Switzerland). According to the published complementary DNA (cDNA) sequence of the chicken p53 (GeneBank accession number: nm205264), specific primers were designed to amplify the DNA-binding domain (DBD) of p53, as shown in Table 2. Using PCR (Polymerase Chain Reaction) techniques, p53 cDNA genes sequences were amplified. The PCR reaction was performed by using a thermal cycler (Takara, Japan) under the following conditions: 95℃ for 5 min, followed by 35 cycles of 95℃ for 30 s, 56℃ for 30 s, 72℃ for 45 s, and a final 72℃ for 10 min extension cycle. DNA templates that were acquired from the MDV positive chickens were amplified using primer pair p53-1/-2. The SPF chickens were regarded as negative controls. DNA fragments were successfully amplified with sizes of 687 bp. The target fragment was gel extracted and connected to the pEASY-T1 vector. Then, the recombinant plasmid was transformed into bacteria and sequencing analysis was made. Finally, the sequencing results were compared with the wild type p53 cDNA sequences reported previously.

**Table 2:** Mutations of p53 genes in MD

		Mutation analysis				
Sample Info		Base mutation sites			Amino acid mutationsSite from to	Mutation area
NC		ND			ND	
MD (C)		611 T-C; 786 A-C; 828 C -G; 1026 A-G			204 Y A 329 E G	Core domain C-terminal domain
MD (C)		786 A-C; 803 g-A; 828 C-G; 864 C-A			268 R H	Core domain
MD (C)		786 A-C; 828 C-G; 864 C-A			ND	
MD (C)		828 C-G; 864 C-A			ND	
MD (C)		487 delete; 539 T-C; 653 T-C; 786 A-C; 828 C-G			163 Stop	Core domain
MD (C)		828 C-G; 864 C-A				
MD (E)		631–677 delete; 864 C-A; 879 C-A; 1026 T-G			210 Stop	Core domain
MD (E)		786 A-C; 828 C-G; 864 C-A			ND	
MD (E)		651 g-A; 864 C-A; 879 C-A			ND	
MD (E)		674 A-G; 786 A-C; 828 C-G; 864 C-A; 878 C-T; 1094 g-A			225 N S 293 T I 365 G D	Core domain C-terminal domain
MD (E)		786 A-C; 828 C-G; 864 C-A			ND	
MD (E)		651 g-A; 701 g-T; 804 C-T; 828 C-G; 864 C-A; 907 g-A			234 R L 303 V M 152 E Y 153 H Y	Core domain C- terminal domain

NC was SPF chicken without diseases; (E) was experimental chicken; (C) was clinical chicken

### Enzyme-linked immunosorbent assay (ELISA) for P53 and P53 antibody

The P53 antigen and antibody levels of chicken were monitored by ELISA (Mlbio, China). In order to eliminate subjective interference, the assessors were blinded to the background of the samples. The detection ranges were 25–850 pg/ml for P53 antigen and 28-1000 pg/ml for P53 antibody. Samples were diluted five time with a special diluent and all processes were implemented in accordance with the instructions. The absorbance (OD) of each sample was finally measured at a wavelength of 450 nm. The sample concentration was calculated depending on the standard curve.

### Statistical analysis

Statistical analyses were conducted with GraphPad Prism (Version 23; San Diego, CA, USA). The differences in the levels of P53 antigen and antibody were analyzed by using the two-tailed Student’s T-test. Statistical significance was designated as *p < 0.05 or ** *p* < 0.01.

## Abbreviations

cDNA: Complementary DNA
DAB: Diaminobenzidine
DBD: DNA-binding domain
ELISA: Enzyme-linked immunosorbent assay
H-E: Haematoxylin and eosin
IHC: Immunohistochemical
HRP: Horse radish peroxidase
PCR: Polymerase chain reaction
PFU: Plaque forming units

## Availability of data and materials

The data supporting the findings are included in the manuscript.
